# State of World Allergy Report 2008: Allergy and Chronic Respiratory Diseases

**DOI:** 10.1097/1939-4551-1-S1-S1

**Published:** 2008-06-15

**Authors:** Ruby Pawankar, Carlos E Baena-Cagnani, Jean Bousquet, G Walter Canonica, Alvaro A Cruz, Michael A Kaliner, Bobby Q Lanier

**Affiliations:** 1 Tresurer, World Allergy Organization, Nippon Medical School, Dept. of Otolaryngology, 1-1-5, Sendagi, Bunkyo-ku, Tokyo 113-8603, JAPAN; 2 Historian, World Allergy Organization, Faculty of Medicine, Catholic University, Santa Rosa 381, Cordoba X 5000 ESG, ARGENTINA; 3 Secretary General, World Allergy Organization, Montpellier Hospital, Av D'Occitanie 273, Montpillier 34090, FRANCE; 4 President, World Allergy Organization, Ospedale San Martino, University of Genova, Pad. Maragliano, L. Go R. Benzi 10, Genova 16132, ITALY; 5 ARIA Scientific Committee, Faculdade de Medicina da Bahia (UFBA), Av. Sta Luzia 379/1501, 40295 050 Salvador - BA, BRAZIL; 6 Immediate Past-President, World Allergy Organization, Medical Director, Institute for Asthma and Allergy, 11002 Veirs Mill Road, Suite 414, Wheaton, MD 20902, USA; 7 Past Member-at-Large, Board of Directors, World Allergy Organization, Mont Del Plaza, 6407 Southwest Blvd, Fort Worth, TX 76132-2777, USA

## 

This activity was supported by unrestricted educational grants from Stallergenes Allergen Vaccines Worldwide, Nestlé Nutrition Institute, and UCB.

**Stallergenes - **http://www.stallergenes.com/nc/en.html

http://www.nestlenutrition-institute.org/en

http://www.ucb-group.com/**and **http://www.todaysallergies.com

## A Message from Professor G. Walter Canonica President, World Allergy Organization

June 2008

Genoa, ITALY

Dear Colleagues:

One of my first duties as President of the World Allergy Organization (WAO) is the pleasure of presenting the State of World Allergy Report 2008. This exciting new WAO initiative, launched at the World Allergy Congress in Bangkok in December 2007, will provide a biennial review of the explosion of allergies around the globe and will consider how the medical, social and economic issues surrounding allergic diseases should be addressed. This first report provides a snapshot of the prevailing situation in 2008 from the perspective of organizations, such as the WAO and the World Health Organization through the Global Alliance against Chronic Respiratory Diseases, which represent allergists and clinical immunologists -- the clinicians at the forefront of attempts to recognize, assess and address this global crisis. The focus of WAO's mandate for the next two years is ***Innovation in Continuity***. WAO will continue to build on its existing programs to further support and develop new research, education and training initiatives in this specialty and to focus attention on the importance of the allergist as the key coordinator of allergy patient care.

The State of World Allergy Report is a call to action for everyone with an interest in allergy to develop a partnership with WAO in a global effort to control and manage allergic diseases on behalf of the world's population.

Yours sincerely,

Professor G. Walter Canonica

President, World Allergy Organization

